# Cooling of a granular gas mixture in microgravity

**DOI:** 10.1038/s41526-024-00369-5

**Published:** 2024-03-22

**Authors:** Dmitry Puzyrev, Torsten Trittel, Kirsten Harth, Ralf Stannarius

**Affiliations:** 1 Department of Nonlinear Phenomena, Institute of Physics, Otto von Guericke University Magdeburg, Universitätsplatz 2, 39106 Magdeburg, Germany; 2 Research Group ‘Magdeburger Arbeitsgemeinschaft für Forschungunter Raumfahrt-und Schwerelosigkeitsbedingungen’ (MARS), Otto von Guericke University Magdeburg, Universitätsplatz 2, 39106 Magdeburg, Germany; 3 Department of Microgravity and Translational Regenerative Medicine, Medical Faculty, Otto von Guericke University Magdeburg, Universitätsplatz 2, 39106 Magdeburg, Germany; 4 Department of Engineering, Brandenburg University of Applied Sciences, Magdeburger Str. 50, 14770 Brandenburg an der Havel, Germany; 5 Institute of Physics, Otto von Guericke University Magdeburg, Universitätsplatz 2, 39106 Magdeburg, Germany

**Keywords:** Statistical physics, thermodynamics and nonlinear dynamics, Scientific data

## Abstract

Granular gases are fascinating non-equilibrium systems with interesting features such as spontaneous clustering and non-Gaussian velocity distributions. Mixtures of different components represent a much more natural composition than monodisperse ensembles but attracted comparably little attention so far. We present the observation and characterization of a mixture of rod-like particles with different sizes and masses in a drop tower experiment. Kinetic energy decay rates during granular cooling and collision rates were determined and Haff’s law for homogeneous granular cooling was confirmed. Thereby, energy equipartition between the mixture components and between individual degrees of freedom is violated. Heavier particles keep a slightly higher average kinetic energy than lighter ones. Experimental results are supported by numerical simulations.

## Introduction

Exploring the behavior of granular gases in microgravity (*μg*) environments holds immense scientific and practical significance. This area of research advances our understanding of physics, engineering, and even space exploration. From the viewpoint of fundamental physics, ensembles of individual macroscopic particles colliding in a manner similar to gas molecules offer unique perspectives on the basic laws of multiparticle physics. Microgravity allows us to observe and analyze pure granular interactions, eliminating the complex influence of gravity. This can lead to breakthroughs in understanding particle dynamics, energy dissipation, and entropy production.

In astronomy and cosmology, understanding the behavior of granular systems sheds light on the formation and dynamics of celestial bodies, such as asteroids, comets, planetesimals, and planetary rings. One can learn a lot about the way these objects evolve and interact. The study of granular gases can also provide valuable insights into energy dissipation as well as energy and heat transfer mechanisms, with manifold implications for the design of efficient applications on Earth and in space. It should be emphasized that microgravity experiments with granular gases can serve as captivating educational tools, inspiring students in physics, space science, and computer vision.

Whereas the majority of previous experiments and theoretical investigations of granular gases were focused on monodisperse systems, more realistic studies have to take into account that such systems in general are polydisperse. Mixtures introduce an additional layer of complexity. One of the fundamental questions is the partition of kinetic energies among the constituents and among their different degrees of freedom. Additionally, one may investigate the emergent behaviors and self-organization in long-term experiments using these mixtures.

Within this study, we plan to extend the analysis of experimental results on granular gases in three dimensions to polydisperse systems. We report experiments and numerical simulations of a bidisperse mixture and compare energy partition, dissipation (granular cooling), and collision statistics observed in microgravity experiments and numerical simulations.

The dissipative character of particle interactions determines the ensemble properties of granular gases: Clustering^1–10^, non-Gaussian velocity distributions^7,11–21^, and anomalous pressure scaling^4,22^ were described. In contrast to a large number of numerical simulations dealing with these systems, there is a comparably small experimental basis, mostly restricted to two-dimensional (2D) systems, e.g., refs. 17–20,23–25. Few 3D experiments in microgravity have been reported, with spherical grains^3,4,26^, ellipsoids^27^, and rods^28–30^. Most experiments were performed with monodisperse systems, providing fundamental insights into the relations between microscopic processes (particle collisions) and ensemble characteristics (granular temperatures and spatial homogeneity), but they lack a typical feature of most of the natural granular gases, viz. the composition of differently shaped and sized constituents.

Several authors have described the behavior of bidisperse and polydisperse mixtures in such gases^31–37^. Garzó and Dufty^31^ investigated theoretically the influences of differences in mass, normal restitution coefficient, size, and compositions for spheres, on the basis of Enskog kinetic theory. In their model, the granular temperature is found to be larger for the heavier particles, in particular when the restitution coefficient is low. The influence of roughness was studied by Santos et al.^33^. They derived parameter ranges for the mass and diameter ratios where equipartition can be expected. Vega Reyes et al.^34,35^ tested theoretical predictions for sphere mixtures using numerical simulations. They also included rotational motion in their analysis. Mixtures of spherical particles with different radii and masses were also simulated numerically by Bodrova et al.^36,37^. Heating and cooling were considered, yet particle rotations were disregarded for simplicity. Larger, heavier particles were found to have a higher granular temperature than lighter ones. The particular distribution of kinetic energies depends on the choice of the contact model in the simulation. Different granular temperatures for different components are quite common in granular matter, they were, e.g., also predicted for vibrated granular fluids^38^. Experimental data for a confirmation of these theoretical predictions are very scarce so far, and essentially restricted to 2D systems. Such a 2D experiment was reported by Feitosa and Menon^39^ for a narrow vertical box filled with spheres of the same size but different materials. They identified the mass density as the relevant parameter for the energy ratio of different species. The elasticity of particles is not important at least for the comparatively hard spheres examined. Nichol and Daniels^12^ reported an energy non-equipartition between small, lighter, and large, heavier disks. The velocity distributions of the two types of disks were identical. Melby^40^ observed bidisperse spheres on a vertically shaken horizontal plate. The problem with 3D systems of spheres is mainly that in order to have enough collisions between the particles, one has to choose a high filling fraction that makes optical analysis very difficult. The problem was solved by Wildman et al.^41^ by using PET as an observation method. Note that the experiments cited here were all performed with permanently excited granular gases. We report experiments with a 3D mixture of rods of the same length but with two different diameters. Statistical data were extracted in the initial heating phase and, more importantly, during free cooling.

Already in the 1980s, Peter Haff^42^ proposed a scaling law for the kinetic energy loss of a homogeneously cooling dense granular gas of frictionless monodisperse spheres. He predicted a time dependence of the form

1 $${E}_{{{{\rm{kin}}}}}(t)=\frac{{E}_{0}}{{(1+t/{\tau }_{{{{\rm{H}}}}})}^{2}},$$

which yields the scaling *E*_kin_ ∝ *t*^−2^ for times *t* ≫ *τ*_H_. *E*_0_ is the kinetic energy at time *t* = 0. The Haff time *τ*_H_(*E*_0_) for a given initial state defines a time scale of energy loss. It depends on material properties and the shape of the grains, and on other system parameters.

Haff’s law was confirmed for freely cooling dilute ensembles of monodisperse rod-like particles^30^, spheres^25,26^, and oblate ellipsoids^27^. Estimates for the mean kinetic energies and *τ*_H_ were obtained. For mixtures, an important and not fully understood question is how the components differ in their kinetic and cooling parameters.

**Fig. 1:**
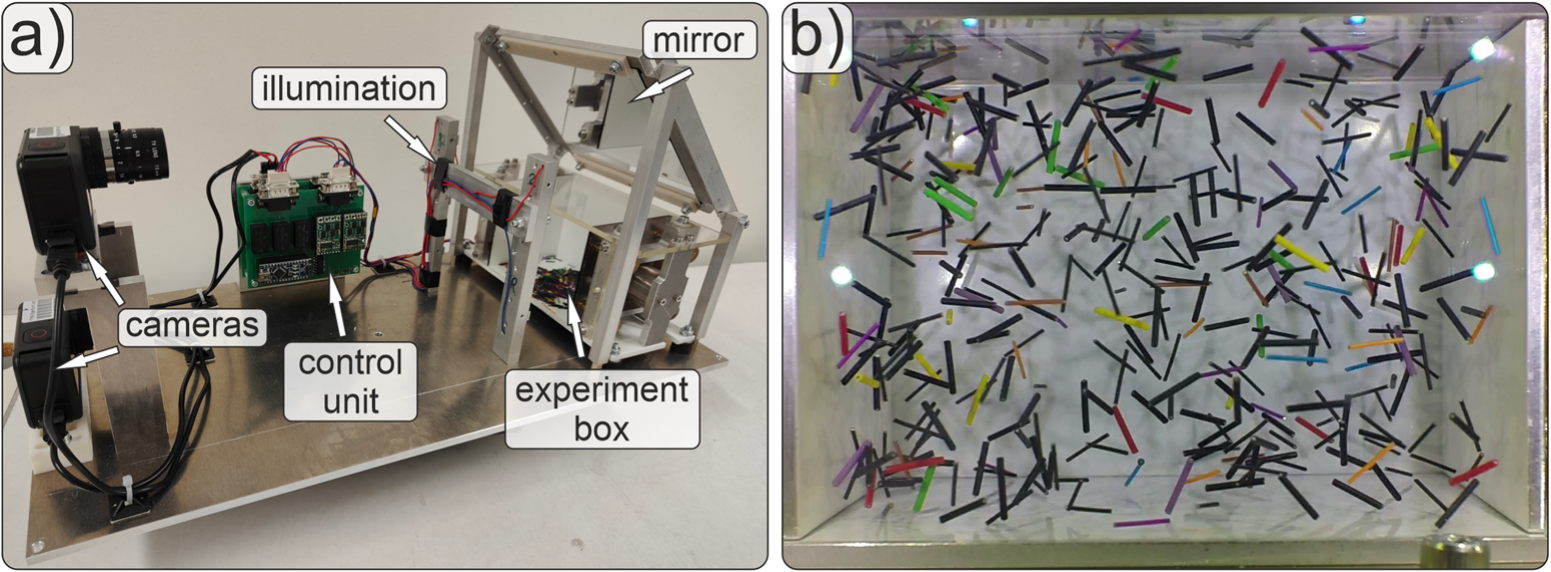
Experimental setup. **a** Photo of the setup. The experiment box with two vibratable side walls contains a mixture of elongated grains, currently lying on its bottom. In *μ*g, they distribute in the container and form the granular gas, which is observed by two cameras through the front and top transparent walls. **b** Snapshot from a video showing a granular gas mixture during cooling in microgravity. The image width is about 12 cm.

In the present paper, we analyze the dynamical properties of a two-component granular gas mixture. Our experimental setup, presented in Fig. 1, is described in detail in ref. 30: Two cameras viewing along axes *y* and *z* were used for a stereoscopic observation of a granular gas containing *N*_1_ = *N*_2_ = 192 rods of each kind (384 rods in total) in a container with dimensions *L*_*x*_ × *L*_*y*_ × *L*_*z*_ = 11.2 × 8.0 × 8.0 cm^3^. The rods consist of insulated copper wire pieces of length *ℓ* = 10 mm and diameters *d*_1_ = 0.75 mm (component 1) and *d*_2_ = 1.35 mm (component 2), respectively. The mass of the thin rods is *m*_1_ = 22 mg, and their moment of inertia for rotations about the long axis is *J*_∥1_ = 0.99 pN m s^2^, and perpendicular to it *J*_⊥1_ = 183 pN m s^2^. For the thicker particles, the mass is *m*_2_ = 37.5 mg, *J*_∥2_ = 4.6 pN m s^2^, and *J*_⊥2_ = 315 pN m s^2^.

The rod mixture was excited during the first two seconds of *μg* along the *x*-axis, and then it underwent granular cooling. The particles were tracked during both the heating and cooling phases. In addition, numerical simulations of the system were performed. For additional details of the experiment and numerical simulations, see the “Methods” section.

## Results

### Cooling rate and Haff time

**Fig. 2:**
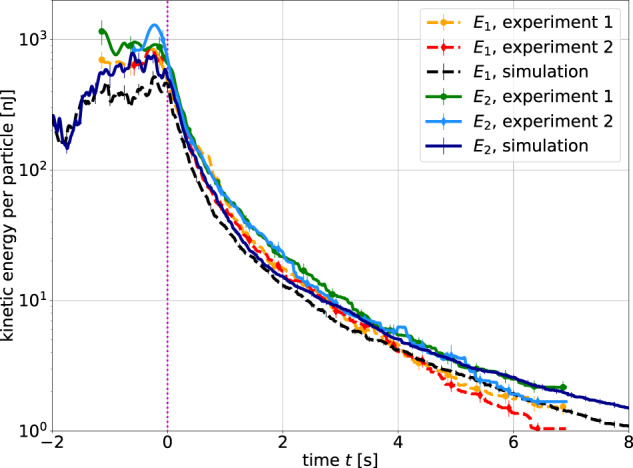
Comparison of the total kinetic energies for the two mixture components in experiment and simulation. Index 1 refers to the thin rods, and index 2 to the thick rods. Time zero refers to the stop of excitation (vertical dashed line), 2 s after entry into the microgravity phase.

**Fig. 3:**
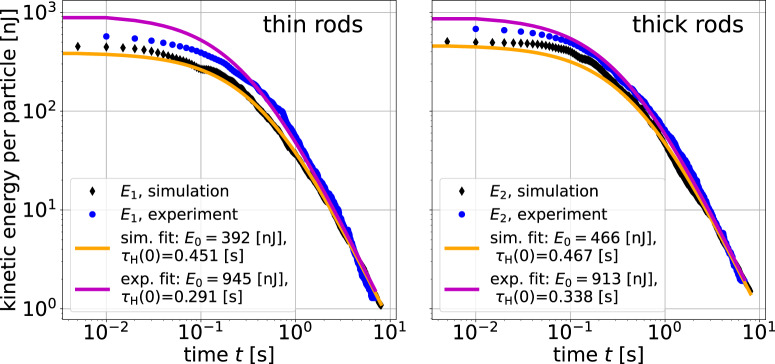
Total kinetic energy for the two mixture components in double logarithmic scale. The energies for both components are fitted well for *t* > 0.2 s with Eq. (1) and the parameters given in the graphs.

Figure 2 shows the decay of the average total kinetic energy per particle separately for the two components, thin (*E*_1_) and thick (*E*_2_) rods. In simulations, the average kinetic energy (granular temperature) for each rod type is calculated as

2 $$E={\overline{E}}_{x}+{\overline{E}}_{y}+{\overline{E}}_{z}+{\overline{E}}_{{{{\rm{Rot\perp }}}}}+{\overline{E}}_{{{{\rm{Rot}}}}\parallel }=\frac{m\overline{{V}_{x}^{2}}}{2}+\frac{m\overline{{V}_{y}^{2}}}{2}+\frac{m\overline{{V}_{z}^{2}}}{2}+\frac{{J}_{\perp }\overline{{\omega }_{\perp }^{2}}}{2}+\frac{{J}_{\parallel }\overline{{\omega }_{\parallel }^{2}}}{2},$$

with respective masses and moments of inertia. The translational velocities *V*_*x*_, *V*_*y*_, *V*_*z*_ are defined with respect to the center of the experiment box. Rotational velocities are defined in the particle-centered system of coordinates, with *ω*_⊥_ corresponding to rotations around the short rod axis and *ω*_∥_ to rotations around the long rod axis. Averaging was performed over all particles of the corresponding type. Following the definition of the granular temperature^43,44^, the velocity of the center of mass of the particle ensemble (velocity field) is subtracted from individual particle velocities before the calculation of average kinetic energies. Note that as in^28,30^, in later stages of heating and during cooling, the velocity of the system is generally small in comparison to average absolute particle velocities. Time is counted from the start of granular cooling after the excitation is switched off. Vertical lines represent the uncertainty that arises mainly from the limited ensemble size of evaluated rods^30^. Of the experimental data, two separate runs (drops) are presented. One observes satisfactory agreement between both experimental runs and the simulation (note that in the experiment, rotations about the long rod axis were not observable and are thus not included in the evaluation). In the subsequent figures, the data for both experimental runs are combined. The mean energies per particle at the end of the heating phase are *E*_1_ ≈ 580 nJ and *E*_2_ ≈ 660 nJ (see Fig. 2). Figure 3 shows that Haff’s law, Eq. (1), well reproduces the loss of kinetic energy during granular cooling for both experimental and simulated data, except for the initial 0.2 s after heating was switched off. This is due to inhomogeneities in the initial heated state^30^, expected to be qualitatively similar to the 2D data for spheres in ref. 24. For this reason, the fit parameter *E*_0_ does not represent the actual initial energy. Most importantly, the fitted Haff times *τ*_H_ differ only slightly for the two components, namely, *τ*_H1_ = 0.45 ± 0.02 s and *τ*_H2_ = 0.47 ± 0.02 s in the simulation, while *τ*_H1_ = 0.29 ± 0.03 s and *τ*_H2_ = 0.34 ± 0.03 s in the experiment. Within the statistical accuracy of our data, this is consistent with equal Haff times for both components. If a slight systematic deviation actually exists, it means that the heavier particles initially cool slower until an equilibrium is reached with equal Haff times and the energy distribution at later stages is slightly shifted further away from equipartition. The reported difference in Haff times between experiments and simulation is not problematic. Note that the Haff time has the property *τ*_H_(*t*_0_ + *t*) = *τ*_H_(*t*_0_) + *t*, so that the difference of the initial Haff times in the experiment and simulation appears as a simple time shift of 0.15 s between the experimental and simulated data (cf. Fig. 2). In Fig. 2, one observes, that the mean kinetic energy of both components at the start of cooling (i.e., for *t* < 0) is smaller in the simulation than in the experiment, which reaches similar mean energy after ≈ 0.15 s

The decay of the average kinetic energies during cooling can also be plotted against the average total number of collisions in the system. Effectively, this scales the time with the collision frequency and can provide a “natural” physical time scale for the homogeneous cooling state. However, this introduces an additional degree of uncertainty into the data analysis due to the collision detection procedure (see the Methods section). Thus, the accurately known time *t* was chosen for plotting in the present paper. In Supplementary Information, we provide a plot similar to Fig. 2, where the kinetic energies are plotted against the total average collision number. It may serve as an additional illustration of the homogeneous cooling of the system according to Haff’s law.

### Energy partition

**Fig. 4:**
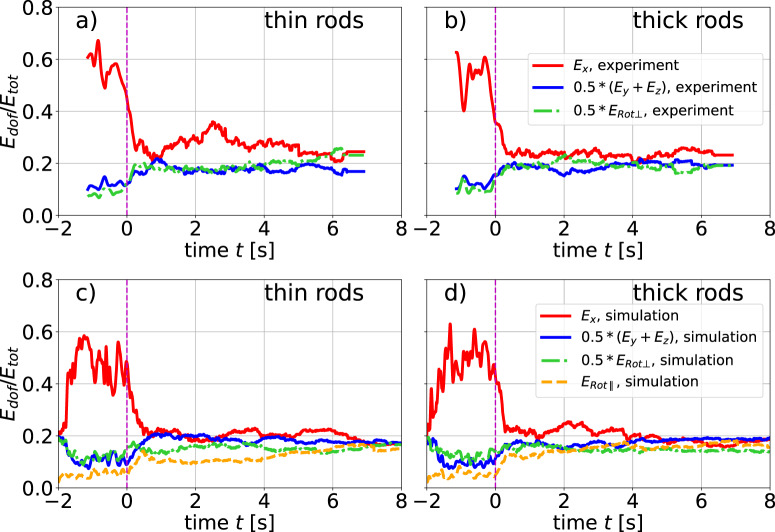
Partition of the mean particle energies per DOF. Panels **a**, **b** refers to the experiment; **c**, **d** to the simulation. Here and in the following figures, vertical dashed lines at *t* = 0 mark the end of the excitation and the start of cooling.

We first compare the shares of kinetic energy per individual degree of freedom (DOF) of each rod type. An excess of translational energy along the excitation direction *x* is observed for both components during heating (Fig. 4). The average energy for the directly excited DOF can reach more than half of the total kinetic energy. This is in accordance with previous findings^16,24,28,39,45,46^ and arises mainly from ‘hot’ particles directly after collisions with vibrating walls. In addition, we find a local packing fraction gradient towards the center of the container in the heated *x*-direction (slightly higher packing fraction near the center), similar to experiments with excited dense granular ensembles^47^ and dilute 2D granular gases^24^. The velocity distribution functions are non-Gaussian. The continuously heated phase was not the primary focus of this study, and the reported properties serve as a characterization of the initial state before cooling only. A thorough analysis of the energy balance in continuously heated systems deserves a more dedicated experiment.

Figure 4 shows the energy partition between DOF associated with translational motion (*E*_*x*_, *E*_*y*_, *E*_*z*_) as well as the two rotational DOF about the short rod axes (*E*_Rot⊥_). After the onset of cooling, the share of energy associated with translation along *x* rapidly decreases. We obtain kinetic energies per DOF which are close to equipartition, except for slight residual dominance of translations along *x*. The energy partition for cooling monodisperse rod ensembles was studied in Refs. 30,48: After a relatively short initial period of approximately three collisions per rod, the particles were found to reach a steady partition of *E*_*x*_, *E*_*y*_, *E*_*z*_, and *E*_Rot⊥_. The average energy associated with the rotational DOF was found around 10-20% less than for translations.

The kinetic energy *E*_Rot∥_ contained in the 6th degree of freedom, rotation around the long rod axis, is quite difficult to determine experimentally, and was not accessible in the present experiments. An approximate value was given in ref. 30, where it was estimated that *E*_Rot∥_ is about one order of magnitude lower than the mean energies of the other DOF. In our simulations, all DOFs are directly accessible. The energy partition extracted from the simulation (Fig. 4c, d) yields energy levels for *E*_Rot∥_ contributing around 10% to the total kinetic energy at the beginning of cooling, and even slightly growing afterward. This is significantly more than the value obtained earlier experimentally^30^. These rotations about the long axis are exclusively excited by frictional contacts. Trusting the experiment, we presume that the realization of these frictional contacts in the simulation is not yet satisfactory and needs refinement.

**Fig. 5:**
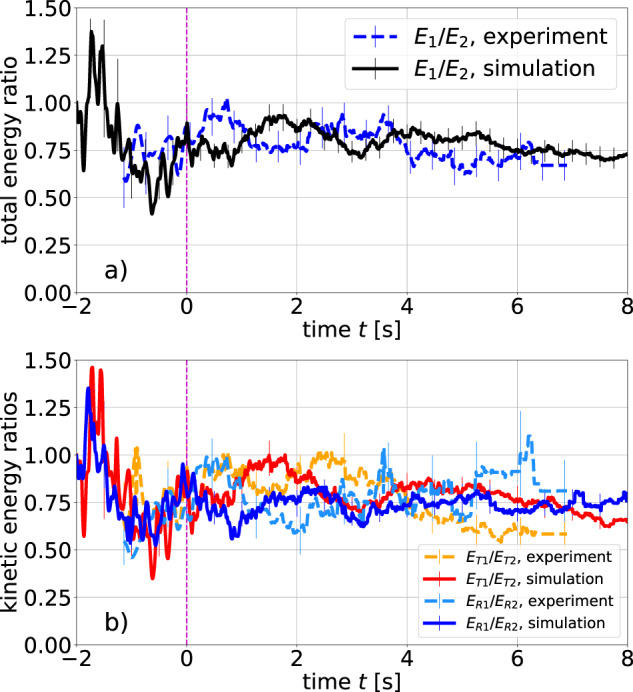
Partition of the kinetic energy between thin and thick rods and between translational and rotational motion. **a** Ratio of the total kinetic energies *E*_1_, *E*_2_ per particle for the mixture components 1 and 2. The vertical dashed line marks the start of cooling. **b** Ratios of translational (*E*_*T*1_, *E*_*T*2_) and rotational (*E*_*R*1_, *E*_*R*2_) kinetic energies per particle for the two mixture components.

A crucial question for granular gas mixtures is the dependence of the mean kinetic energy on particle properties such as relative particle size and mass. In our experiments, the mass ratio is *m*_1_/*m*_2_ = 0.59 (diameter ratio *d*_1_/*d*_2_ = 0.56). An excess of the average kinetic energy of the larger particles persists during the complete cooling phase, as seen from the ratio *E*_1_/*E*_2_ ≈ 0.8 of the average total (observed) kinetic energies per particle for the mixture components in Fig. 5a, both during the heating and cooling stages.

Figure 5b shows the ratios of translational (*E*_*T*1_/*E*_*T*2_) and rotational (*E*_*R*1_/*E*_*R*2_) energies separately. The data are more noisy than the total energy due to the permanent energy exchange between translational and rotational DOF. Nevertheless, we observe satisfactory statistical agreement between the experiment and simulation.

### Collision statistics

**Fig. 6:**
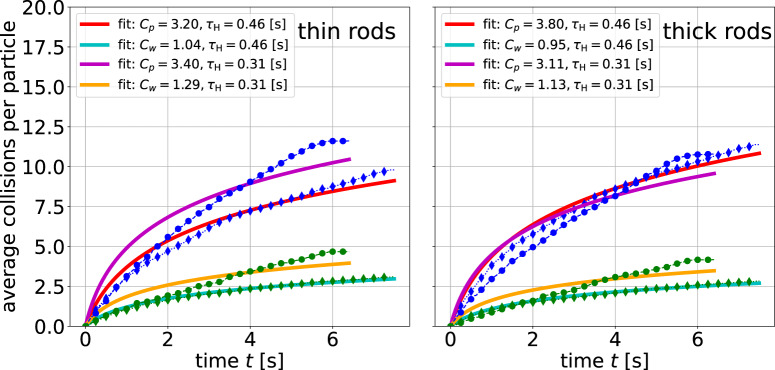
Cumulative statistics of particle-particle (blue markers) and particle-wall (green markers) collisions for thin and thick rods. Solid lines fit with logarithmic functions of the form given in Eq. (3). Double collisions of the rods with a wall^49^ count as one (see text). The Haff times were held fixed.

**Fig. 7:**
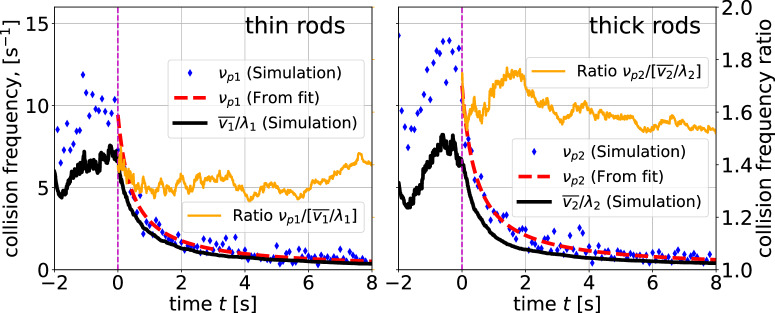
Average particle-particle collision frequencies *ν*_*p*1,2_ for the thin and thick rods. The orange lines show the ratio of the actual collision frequency retrieved from the simulations to the one obtained theoretically from the mean velocity and the mean free path estimate.

The statistics of collisions as the elementary steps of the cooling process are compared in Figs. 6 and 7. Figure 6 shows the cumulative number of collisions of each particle type with other particles and with the container walls, extracted directly from the simulations, as well as from an analysis of experimental particle trajectories. We note that collisions with the container walls often involve two contacts^49^, one with each of the rod ends. Such double contacts are counted as one. Following Haff’s model, the average cumulative number of particle-particle collisions in the system can be approximated by^30,45,50^

3 $${N}_{{{{\rm{C}}}}}(t)={C}_{p}\ln \left(1+\frac{t}{{\tau }_{{{{\rm{H}}}}}}\right),$$

where time *t* starts with the beginning of cooling. Here, *C*_*p*_ is a positive constant related to the mean energy loss in a single particle-particle collision^30,50^. Since the number of collisions is the ratio of the mean speed of the particles and some time-independent geometric parameter (i.e., the mean free path) both particle-particle and particle-wall collisions should obey the same logarithmic dependence as in Eq. (3), with different prefactors *C*_*p*_ and *C*_*w*_. The four curves in Fig. 6 were fitted by Eq. (3) with mean initial Haff times *τ*_H_ = 0.31 s (experiment) and 0.46 s (simulation). While the simulation collision numbers are accurate and match the fits quite nicely, the experimental results differ significantly. The possible reason is that not all collisions could be correctly identified in the videos with our automated collision detection, both in the initial phase where particles move too fast, and in the later stages of cooling where noise in the detected trajectories can be mistaken for collisions. A more accurate collision detection would be possible in experiments with somewhat lower packing fractions and an improved camera setup. Nevertheless, it is evident that particle-wall collisions are around 2.5 − 3 times less frequent than particle-particle ones for the thinner rods and around 3.5–4 times less frequent for the thicker rods.

For comparison, we modified the formula for the collision cross-section of rods^30^ to include cylinders of different diameters. A random particle orientation respective to the flight direction and a uniform, homogeneously mixed particle distribution are assumed for simplicity. We obtain estimates of the mean free paths *λ*_1_ and *λ*_2_ as:

4 $${\lambda }_{i}=\frac{V}{\sqrt{2}}\left(\frac{1}{{N}_{1}{\sigma }_{1i}+{N}_{2}{\sigma }_{2i}}\right),\quad (i,j=1,2,),$$

where the scattering cross sections *σ*_*ij*_ corresponding to collisions of particle types *i* and *j* can be approximated as:

5 $${\sigma }_{ij}=\frac{\pi {\ell }^{2}}{8}+\left(\frac{3+\pi }{4}\right)\left({d}_{i}+{d}_{j}\right)\ell +\frac{9{d}_{i}{d}_{j}}{2\pi }+\frac{{d}_{i}^{2}+{d}_{j}^{2}}{2}.$$

The estimated mean free paths are *λ*_1_ = 1.92 cm and *λ*_2_ = 1.66 cm, resp., in the absence of wall collisions. A rough estimate of the mean free path of a sphere in a cubic container with sides *ℓ* yields *λ*_w_ ≈ 0.583*ℓ*, which for our experiment is roughly 2.7*λ*_1_ or 3.2*λ*_2_. Thus, the predicted ratio of collisions with particles and with walls should be around 3, in fairly satisfactory agreement with the experiment.

Another detail of the collision statistics is elucidated in Fig. 7. When the particle-particle collisions counted in the simulation are compared to the expected value of $\bar{v}/\lambda$ with the mean particle velocity ${\bar{v}}_{1,2}$ and the mean free paths *λ*_1,2_ from Eqs. ((4),(5)), a factor of ≈ 1.5 is evident. The mean free paths directly extracted from the simulations are also ≈ 1.5 times shorter than their theoretical estimates. A possible reason is that the simplified collision model considers only non-rotating rods. Fast rotations about the short axes obviously increase the scattering cross sections.

## Discussion

Summarizing, a quantitative confirmation of the main features of the granular cooling of a bidisperse mixture of rods was achieved here. Heavier particles have larger mean energies both during excitation and during cooling. The ratios *E*_1_/*E*_2_ of the average kinetic energies per particle were around 0.8 both in experiment and simulation (with the mass ratio being 0.59 and the moment of inertia ratio 0.58 for the rotations about the short axes). Even though a direct comparison with theoretical predictions for spheres in the literature^31,39^ may not be useful because of the fundamental differences between both systems and the neglect of rotational motion in the theoretical model, it is satisfying that the results agree at least qualitatively. The heavier particles carry more kinetic energy than the lighter ones. Effects of roughness that were described in analytical and numerical studies^33,51^ are much less relevant for rods, where the torque and the exchange of rotational and translational energies during collisions depend to a large extent on the position of the contact points along the rod axes.

The ratios of kinetic energies of both species that prevail in the cooling phase are established already during the excitation, after approximately 1 second. Within this time interval, particles have experienced between 5 and 10 collisions among each other (see Fig. 7). This is at least qualitatively consistent with ref. 41 where a similar partition was measured during permanent excitation of bidisperse spheres, even though a quantitative comparison of rod and sphere systems may not be appropriate.

The Haff time determined for heavier particles was found to be slightly larger than that of the lighter ones, which might indicate a slower cooling of the heavier particles until an equilibrium *E*_1_/*E*_2_ is reached, that is smaller than the value reached within our observation time scale. In perspective, this study shall be extended to bidisperse mixtures of particles that are significantly different from each other^52^, particularly with different shapes. The investigation of polydisperse mixtures is highly desired, but several problems including the automatic detection and distinction of components need to be solved first.

In experiments with excited granular gases under gravity, convective vortices have been reported^53,54^. This is, however, a completely different situation from our experiments since both the gravitation direction as well as the peculiar sidewall structure^54^ break the up-down symmetry in those experiments. Convective rolls were also predicted theoretically^55^ in a 2D cell under microgravity. We have not found signs of such structures. One reason may be the limited observation time compared to the growth rate of the convection amplitude. For the same reason, there are no significant spatial inhomogeneities that could be regarded as clusters.

## Methods

### Experimental setup and data analysis

The experiment was performed in the ZARM drop tower in Bremen, where microgravity is achieved for about 9.2 s. The quality of the microgravity during this time period is excellent, better than 10^−6^*g*. Initially, the system was excited by sinusoidal vibration of two side walls of the container (in *x*-direction) at an amplitude of *A* = 0.24 cm and frequency *f* = 30 Hz, which corresponds to a maximum plate acceleration of ≈ 8*g*. Then, the vibration was stopped and the granular gas mixture was left without energy input in a granular cooling phase.

3D particle detection in the experiments, tracking, and trajectory post-processing mainly follow the outlines of refs. 8,30. The ensemble was observed with two video cameras *GoPro Hero 3 Ribcage*. The image resolution is 1280 × 960 pixel^2^ at a frame rate of 100 fps. For easier detection and tracking, 48 rods of each type have a colored surface, and the remaining 144 non-tracked particles of each of the two types are black and serve as a ‘thermal’ background (the color does not affect the mechanical properties of the rod). Altogether, eight different colors were available with 12 particles of each color. The choice of 96 colored particles in each experiment is near the limits of the detection and tracking feasibility with the current setup and packing fraction.

For particle detection and tracking, we followed the workflow described in ref. 9. The program was significantly improved, transferred to the Detectron 2 framework^56^, and a custom graphical user interface (GUI) was added for preview and correction of 2D and 3D data.

A data set containing camera image frames together with the corresponding rod endpoints was assembled, first manually, and later with an iterative procedure for corrected rods. The current data set includes around 2500 images (around 300,000 object instances of colored rods) from both camera views of several independent runs of different experiments. Data processing scripts and pre-trained Detectron 2 network model files as well as the GUI program for correction and annotation of data are being prepared for publication as an open-source software package.

In order to track the colored rods between the frames, the rod endpoints are triangulated and matched by solving the 3D axial optimal assignment problem (also known as bipartite graph matching). We found that an optimization towards both the reprojection error for rod endpoints and the displacement of the rod endpoints between frames is required for robust tracking of the particles.

The rod trajectories were fitted taking into account constant translation velocities and rotation rates during the free flight phases of the particles between the collisions. For the rod centers of mass, the *l*_1_ trend filtering optimization method^57,58^ was used. It allows to fit the noisy particle center trajectories to a sequence of piecewise linear functions with kinks (bends of the fitting function) in between.

We extracted angular velocities by differentiating the rod orientation quaternions^59–61^. For the smoothing of high-frequency noise due to the orientation measurement error, a moving average filter was applied. Then, the resulting angular velocity data were fitted with a similar trend filtering approach as the translations, where instead of the piecewise linear approximation, we fitted the angular velocities with piecewise constant (step) functions.

For translational as well as angular velocities, we employed the special case of the *l*_1_ trend filtering for vector time series as described in^57^. The advantage of overfitting the *x*, *y*, *z* center coordinates together is that the fitted coordinate components tend to show simultaneous trend changes at common kink points, which correspond to changes in velocities due to collisions of the particle. One problem that arises when applying this procedure is that as particles slow down, the relative weight of the kinks in the optimization procedure decreases and the standard *l*_1_ trend filtering procedure tends to overestimate the number of collisions. To improve the fitting, we have employed iterative weighted heuristics from Kim et al.^57^, which allows us to optimize the fit towards the number of kinks instead of the sum of their residual norm.

We assume that the optimization error that arises at the bending points of piecewise linear approximations of particle endpoint velocities as well as jumps in angular velocities signalize that the particle collided with another particle or the wall. Then, knowing the positions of the walls, collisions can be attributed to either particle-particle or particle-wall collisions. This way, the collisions undergone by rods can be determined automatically, even though the absolute precision may not always be satisfactory.

### Numerical simulations

In order to support the experimental analysis, we performed a numerical simulation. It provides the opportunity to extract particular properties that are not accessible in the experiment. At the same time, a comparison of the simulation results with the experiment helps to choose a suitable collision model and realistic material parameters. It should be noted that a completely accurate simulation of our experiment is an extremely hard task, which might not even be feasible, due to multiple factors such as slightly different shapes of individual rods, the number of parameters in contact models, and the overall complexity of particle collisions. At the same time, reasonable statistical agreement between the experiment and simulations can be achieved and analogous system behavior can be demonstrated in both cases, as demonstrated for the excitation phase^46^.

We used a hybrid graphics and central processing unit (GPU-CPU) implementation of discrete element modeling (DEM)^46,61–64^, adapted to systems with moving walls. The collision detection and nonlinear Hertz-Mindlin contact force model follow the previous simulations with spherocylinders^9,46,64^. Our software was modified to simulate bidisperse mixtures of particles. The model considers the dynamics of an ensemble of two types of spherocylinders, with the same length *ℓ* and radii *r*_1_ and *r*_2_ as in the experiment, i.e., aspect ratios *ζ*_1_ = *ℓ*/(2*r*_1_) and *ζ*_2_ = *ℓ*/(2*r*_2_).

Contact detection between two particles reduces to finding the minimal distance between line segments that correspond to the axes of two cylinders. For the particle-wall collisions, we used the same model but assumed the interaction of a spherocylinder with an infinite static or moving plane. The force ${\overrightarrow{F}}_{ij}$ exerted on particle *i* by particle *j* reads as: ${\overrightarrow{F}}_{ij}=-{\overrightarrow{F}}_{ji}$, and it can be decomposed as ${\overrightarrow{F}}_{ij}={F}^{{{{\rm{n}}}}}\cdot \overrightarrow{n}+{F}^{{{{\rm{t}}}}}\cdot \overrightarrow{t},$ where *F*^n^ is the component normal to the contact plane and *F*^t^ acts in the tangential direction, $\overrightarrow{n}$ and $\overrightarrow{t}$ are the respective unit vectors. Here, *F*^n^ was modeled as a Hertz-type force^65^, depending on the overlap distance *δ* between two spherocylinders. The energy loss was quantified using an effective restitution coefficient *e*_*n*_ = 0.7. The friction coefficients for particle-particle and particle-wall collisions were varied between at *μ* = 0.2 and *μ* = 0.8, with *μ* = 0.4 chosen for the presented set of simulations. While the real values of restitution coefficient and friction are not known, the chosen values of *e*_*n*_ and *μ* provide reasonable agreement with the experiment, even though the computed Haff times are somewhat larger. The Young modulus was set to *Y* = 5 GPa, yet its particular value does not influence the results noticeably as long as it is large enough to restrict excessive particle overlap, and small enough so that the collisions are resolved in a sufficient number of simulation steps (*δt* = 5 × 10^−8 ^s)^66^. A velocity Verlet numerical algorithm^67^ was used to integrate the 3D translational equations of motion of each particle, while the rotational motion of particles was resolved using a modified leap-frog algorithm^68^. Container and excitation parameters were the same as in the experiment.

### Reporting summary

Further information on research design is available in the Nature Research Reporting Summary linked to this article.

### Supplementary information

Supplemental Information

Reporting Summary

## Data Availability

The source experimental data (images from two drop tower experiment runs) are publicly available at 10.5281/zenodo.10556253.

## Code availability

Particle detection, tracking, and trajectory correction code are publicly available at https://github.com/ANP-Granular/ParticleTracking. The numerical simulation code and additional data processing scripts are planned for open-source publication and are currently available from the corresponding author upon request.
