# Supplementary material for: Cooling of a granular gas mixture in microgravity
Source: NPJ Microgravity. 2024 Mar 22;10:36. doi: 10.1038/s41526-024-00369-5 (PMC10959983; doi:10.1038/s41526-024-00369-5)
Supplement: Supplementary file 1 — Supplemental Information [file 41526_2024_369_MOESM1_ESM.pdf]

Cooling of a granular gas mixture in microgravity

Dmitry Puzyrev[1,2,3*], Torsten Trittel[4,1,2,3], Kirsten Harth[4,1,2], Ralf Stannarius[1,2,4,5]

[1]Department of Microgravity and Translational Regenerative Medicine, Medical Faculty, Otto von Guericke University Magdeburg, Universitätsplatz 2, Magdeburg, 39106, Germany.
[2]Research Group 'Magdeburger Arbeitsgemeinschaft für Forschung unter Raumfahrt-und Schwerelosigkeitsbedingungen' (MARS), Otto von Guericke University Magdeburg, Universitätsplatz 2, Magdeburg, 39106, Germany.
[3]Department of Nonlinear Phenomena, Institute of Physics, Otto von Guericke University Magdeburg, Universitätsplatz 2, Magdeburg, 39106, Germany.
[4]Department of Engineering, Brandenburg University of Applied Sciences, Magdeburger Str. 50, Brandenburg an der Havel, 14770, Germany.
[5]Institute of Physics, Otto von Guericke University Magdeburg, Universitätsplatz 2, Magdeburg, 39106, Germany.

*Corresponding author(s). E-mail(s): dmitry.puzyrev@ovgu.de;

Supplementary Notes

Alternative to presenting the decay of kinetic energies E_1, corresponding to the thinner rods, and E_2, corresponding to the thicker rods, in real time scale as provided in **Fig. 2** in the main manuscript, one can plot the same quantities in a "natural" time scale of the system, which is defined by the average cumulative number of collisions experienced by the particles.

Supplementary Figure 1 shows the average kinetic energies for both particle types in this adjusted time scale, where the horizontal axis corresponds to the average total (particle-particle and particle-wall) cumulative number of collisions N_{C} in the system, averaged over all particles. For better comparison with the experimental time,

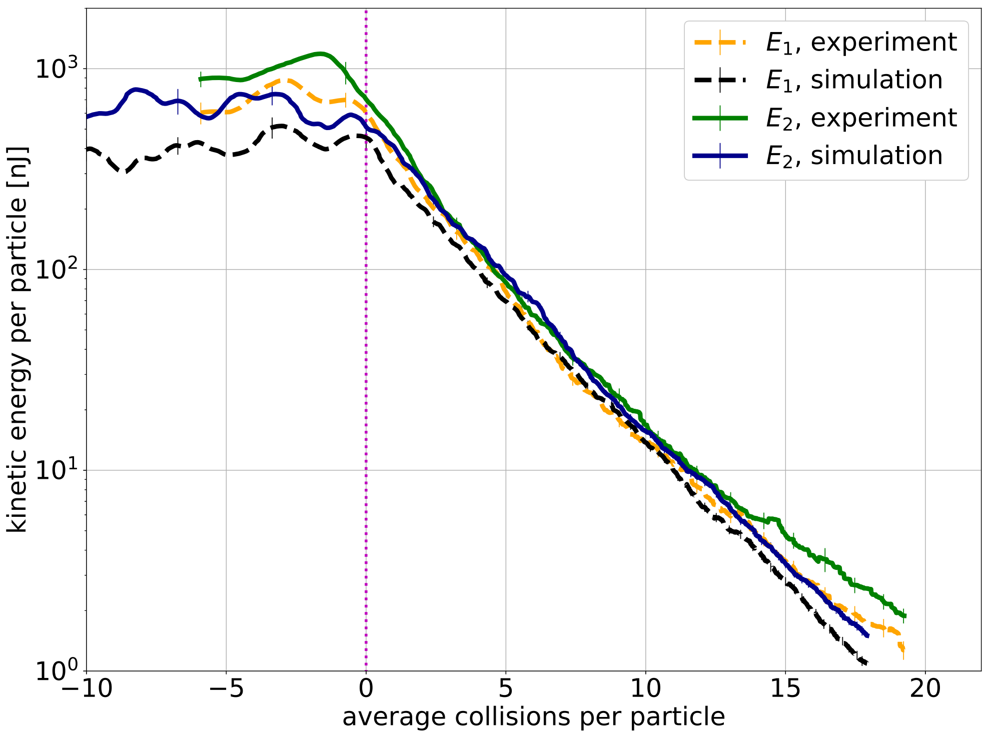

Supplementary Fig. 1 Total kinetic energy for the two mixture components from experiment and simulation in comparison. The abscissa gives the mean number of collisions per particle minus the mean total number of collisions during heating. Zero thus refers to the stop of excitation (vertical dashed line), 2 s after entry into the microgravity phase, E_1 to the thinner and E_2 to the thicker rods.

we subtracted the total number of collisions per particle during heating, so that $N_\mathrm{C} = 0$ always corresponds to the start of the cooling phase. Both simulation and experimental data are presented.

Shortly after the beginning of cooling (after 1-2 collisions), the logarithm of the average kinetic energy decreases linearly with the average cumulative collision number, in accordance with Haff's law. Moreover, the curves remain mostly parallel, which signifies that the system is indeed in a homogeneous cooling state. A consistent shift between the energy curves is observed between thin and thick particles, corresponding to the established ratio $E_1/E_2 \approx 0.8$. This is true for both simulation and experimental data for the major part of the cooling stage. Towards the end of the observation period, a slight diversion from the logarithmic decay is observed for the experimental values.
